# Telling it like it is

**DOI:** 10.1186/2050-5736-1-4

**Published:** 2013-04-25

**Authors:** Adam Shaw, Gail R ter Haar

**Affiliations:** 1 Acoustics and Ionizing Radiation Division, National Physical Laboratory, Teddington, TW11 0LW, UK; 2 Joint Department of Physics, Institute of Cancer Research, Sutton, SM2 5NG, UK

## 

With the amazing successes that have been reported over the past 10 years, it is easy for researchers in therapeutic ultrasound to be seduced by the appeal of biological and clinical studies - but understanding the biology alone is insufficient. All good science requires repeatability, and developing a successful therapy is no different. It is imperative from both a scientific and a commercial perspective that we can not only demonstrate the interaction of ultrasound with biological systems but also quantify the relationship between the delivered ultrasound and the biological effect. Moreover, we then need to be able to control the delivered ultrasound - the ultrasound dose, if you like - to achieve consistent and repeatable treatments.

A distinguishing feature of our new journal - whose stated goal is to ‘accelerate the adoption of therapeutic ultrasound as a clinical tool’ - should be an insistence that best practice is followed first when planning the ultrasound exposure regime and again when reporting those conditions. We should set a clear example for other journals, and for funding bodies, to follow. This will make it apparent, even to those researchers who are not primarily interested in physics and engineering, that knowledge and control of the delivered ultrasound is not something that can be ignored until writing the final paper. It absolutely must be built into a study even before the proposal is submitted. Too often, even in high-quality and esteemed journals, the exposure description is inadequate, and assumptions (for instance, about the mechanism of interaction) are not pro perly explained. The researchers themselves may often not be fully aware of these weaknesses in their study.

It is our belief that a handful of simple guidelines [1] published by the authors of this piece will serve to remind researchers of the importance of good ultrasound measurement and exposure estimation and will lead over time to a greater body of research which can be replicated and tested by others. Our starting principles in preparing these guidelines were simple enough: firstly, that for a quantitative exposure-response study, there must be some measurements of the actual ultrasound field or fields used; and secondly, that the measured values (usually in water, following methods described in international standards) must be reported, and the methods for estimating any other *in situ* or exposure quantities must be clearly laid out. Of course it is true that the ultrasound field measured in water may be very different from the field in tissue or the sample holder *in vitro*. Nevertheless, having a proper description of the undisturbed field is the first step to understanding the experimental conditions and to being able to replicate the exposure. Taken together, these two principles will help avoid many of the potential misunderstandings that can arise.

The full set of recommendations and example wording are discussed in detail in [1]. As well as reading these, authors and reviewers for our journal should ask themselves some simple questions at all stages of their work.

Q1. Does this study attempt to demonstrate correlation between exposure to ultrasound and a physical or biological effect? If it does, it must include acoustic output measurements of the ultrasound field to determine maximum values in the field or, preferably, the acoustic pressure and intensity distributions.

Q2. Are the measured values of output power, acoustic pressure, intensity, and other acoustic quantities reported? Have the measurement methods been described in sufficient detail to be replicated by others?

Q3. Has the temperature rise in the region of the cells or tissue of interest (or in a suitable substitute) been measured and reported, even if a thermal mechanism is not being tested?

Q4. If estimated values of *in situ* acoustic pressure or intensity are reported (for instance by using a ‘derating’ factor), has the method for calculating the *in situ* value been fully explained, and a worked example given? Is the estimated transmission loss in the propagation path reasonable?

Q5. If a thermal or mechanical index is calculated for use as an exposure quantity, has a reasonable value for the attenuation coefficient of the propagation path been used as the derating factor? Has the calculation been stated mathematically and a worked example given?

Asking - and answering! - these questions will help produce a rigorous study which will provide a solid basis for conclusions and for future work. Let us start as we mean to go on with our own journal and demand the highest standards of measurement and reporting of ultrasound fields and exposure levels.

## Competing interests

The authors declare that they have no competing interests.

## Authors’ contributions

Both authors wrote, read and approved the final manuscript.

## Authors’ information

Adam Shaw is a Principal Research Scientist at the National Physical Laboratory. He is a member of the Safety Group of the British Medical Ultrasound Society, sits on two IEC Technical Committees, is Convenor of the IEC Working Group on Exposure pmeters for Ultrasound and is leading an international project to develop standards for ultrasound exposure and dose to tissue. His research interests are in measurement and standardization for ultrasound therapies, in the safe use of medical ultrasound, and in the Primary Standard measurement of ultrasound power. He has published more than 30 papers and three book chapters.

Gail ter Haar is a Physicist, with a DSc in clinical medicine. She is head of therapeutic ultrasound at the Institute of Cancer Research, and Visiting Professor of Therapeutic Ultrasound, Oxford University. Her interests are the development of therapeutic applications of ultrasound for cancer (especially high intensity focused ultrasound, HIFU) and the safety of diagnostic ultrasound. She was founder President of the International Society for Therapy Ultrasound (ISTU), is Scientific Secretary of the European Society for Hyperthermic Oncology, chair of both the European Committee for Ultrasound Radiation Safety and the Safety Group of the British Medical Ultrasound Society. Gail is an honorary member of BMUS, honorary fellow of the American Institute for Ultrasound in Medicine, and fellow of the Acoustical Society of America and IPEM. She is Deputy Editor of “Ultrasound in Medicine and Biology”. She has written more than 160 papers and 28 book chapters.
